# Use of assisted reproductive technologies before and after the Artificial Reproduction Act in Taiwan

**DOI:** 10.1371/journal.pone.0206208

**Published:** 2018-11-01

**Authors:** Jason C. Hsu, Yu-Chi Su, Bo-Yun Tang, Christine Y. Lu

**Affiliations:** 1 School of Pharmacy and Institute of Clinical Pharmacy and Pharmaceutical Sciences, College of Medicine, National Cheng Kung University, Tainan, Taiwan; 2 School of Medicine, College of Medicine, National Cheng Kung University, Tainan, Taiwan; 3 Department of Population Medicine, Harvard Medical School and Harvard Pilgrim Health Care Institute, Boston, Massachusetts, United States of America; Johns Hopkins University School of Medicine, UNITED STATES

## Abstract

**Background:**

Low birth rates and infertility are growing concerns for many countries. The Artificial Reproduction Act (ARA) was implemented in 2007 to better support the use of assisted reproductive technology (ART) in Taiwan. This study aims to examine trends in the use of ART and to determine impacts of the ARA on ART use.

**Method:**

This study used data from the Health Promotion Administration in Taiwan. We used auto-regression models to examine the recent trends (1998–2017) in fertility related indicators and the use of ART. An interrupted time series design and segmented linear regression models were applied to analyze the changes in number of ART treatment cycles and birth rates by ART following the ARA.

**Results:**

The number of births by ART has gradually increased, with an annual growth rate of 21.55%. The rate of births by ART increased from 0.86% in 1998 to 1.44% in 2007, and to 4.33% in 2016 (annual growth rate: 41.23%). We estimated a relative increase of 78.51% (95%CI: 46.13%, 110.90%) in the number of births by ART and a relative increase of 35.67% (95%CI: 18.87%, 52.47%) in rate of births by ART 5 years following the ARA was implemented.

**Conclusion:**

ART has been seen as an approach to improve fertility. Our analysis suggests that the ART use and births associated from ART use both increased in Taiwan following the implementation of ARA. This experience in Taiwan may offer important lessons for other countries that are facing low fertility challenges.

## Introduction

The issue of low fertility rates has been gaining increasing attention in many countries in recent years, including Taiwan. The total global population grown gradually in the past few years, and it has been predicted that it will reach 9.7 billion by 2050.[1] Nearly half of the world’s population is living in countries with low-fertility rates.[2] The national birth rate was only 1.09% in 2014 in Taiwan. In addition, the average age of mothers at first childbirth increased from 26.4 years-old in 1999 to 30.5 in 2014; nearly 70% of mothers at the time of first childbirth were over 30 years-old in Taiwan in 2012. [3, 4] Late marriage and late childbirth might lead to infertility or low fertility, which are not only a burden for individuals but also for society because of demographic changes (aging society) and the associated economic burden (a decreasing proportion of labor force and increasing dependency ratio). [5] Assisted reproductive technology (ART) has become an option to improve fertility rate in recent decades. [6, 7] Late marriage and attempting to get pregnant when older have been the main causes of low fertility rates and infertility [8, 9], and ART has been expected to help improve fertility. ART includes In vitro fertilization (IVF), Zygote intrafallopian transfer (ZIFT), and Gamete intrafallopian transfer (GIFT), among others.

Many countries have developed policies and strategies to address low fertility rate.[10, 11] Particularly, strategies to regulate ART use and enhance the availability of ART have been possible solutions. Previous research examined the contribution of ART to total fertility rate and population structure in UK and Denmark over time.[12] It found that “if access to ART in the UK were increased to the level of Denmark, the total fertility rate would increase by 0.04, from 1.64 to 1.68.”. The results demonstrate that ART has the potential to contribute to total fertility rate.

In view of the traditional cultural values of the Taiwanese society, fertility is the vocation of women. Under such an environment, women are often exposed to responsibilities and pressure when infertility occurs. With the advances in reproductive medicine, ART began to be used by couples with infertility problems, but ART use in Taiwan has always raised issues in ethics, life value, and law. The Taiwan government regulations on the implementation of ART alleviate the aforementioned ethical issues and protect the reproductive rights (except the use of “surrogate mother”, is not accepted by traditional social values in Taiwan). In 2007, the Artificial Reproduction Act [13] (excluding the surrogate mother issues) was finalized and enacted. Prior to the 2007 ARA, the government did not provide subsidies for artificial reproduction, which are associated with enormous expensive medical expenses that couples with infertility problems must bear out-of-pocket costs.

According to the Artificial Reproduction Act [13], a medical care institution is allowed to perform artificial insemination for a married couple once all the following conditions are satisfied: (1) health assessment before receiving artificial insemination; (2) the husband or wife of the married couple has been diagnosed as suffering from infertility, or has been diagnosed as suffering from a major hereditary disease, and it is suspected that natural conception and birth would produce abnormal children; (3) at least one member of the married couple has healthy reproductive cells, and that person has no need to accept donated sperm or oocytes. In addition, medical care institutions are required to provide information concerning the donor’s ethnicity, skin color, and blood type for the reference of the recipient couple. However, donors are not allowed to specify recipients, and vice versa. Furthermore, any of the circumstances or methods prescribed in the following subparagraphs is prohibited for artificial reproduction: (1) sperm and oocytes from direct blood relatives, direct relatives by marriage and collateral blood relatives within the forth degree of kinship; (2) using reproductive cells or embryos provided exclusively for research purposes; (3) creating a human embryo other than by fertilization; (4) selection of the embryo’s sex; this restriction shall not apply, however, when there is a reason connected with hereditary disease; (5) mutual donation of sperm and oocytes; (6) using an embryo cultured in vitro for more than seven days; (7) implantation of more than five embryos at a time; (8) use of mixed semen, and (9) use of donated reproductive cells imported from outside the country. The act was intended to improve the use of ART, to protect rights of couples with infertility problems, and to enhance donations required for artificial reproduction. Although ART is not financially covered by Taiwan’s government (the National Health Insurance System), low-income families can apply for allowances from the government, and many district governments have also provided various different allowances.

The development of ART may have improved the national fertility rate. However, little is known about the trends in fertility rate and use of ART in Taiwan. The objective of this study was to examine trends related to use of ART and birth rates using ART before and after the 2007 Artificial Reproduction Act.

## Method

### Data sources

We obtained 1998–2016 (18 years) data related to use of ART in Taiwan from “The Annual Report on the Performance of ART.” [4] The data include yearly number of ART treatment cycles and yearly number of births related to ART. We also obtained 1998–2017 yearly population data by region from the Department of Statistics, Taiwan Ministry of Interior. [3] It covers the yearly number of people by age, gender, and marital status; national annual number of births; average female married age; and average mother’s age at first childbirth.

### Measures

We obtained and calculated the following variables: (1) rate of unmarried people aged over 15: number of unmarried people aged over 15 divided by national population aged over 15 (not including divorced and widowed people); (2) average female married age; (3) average mother’s age at time of first childbirth; (4) national birth rate, calculated by number of births divided by national population aged over 18 in the same year; (5) number of ART treatment cycles; (6) number of births by ART: number of births by ART divided by national number of births in the same year; (7) national number of births and (8) rate of births by ART: number of births by ART divided by national number of births.

### Statistical analysis

To examine the recent trends (1998–2016) in use of ART, we applied a time series design with an autoregressive model to calculate the annual growth rates for the measurements referenced above. [14]

To understand the impact of the 2007 Artificial Reproduction Act on the Use of ART, we used an interrupted time series design and segmented linear regression models. We assessed the changes in “number of ART treatment cycles,” “number of births by ART,” and “rate of births by ART” following the 2007 Artificial Reproduction Act. Since the effect of the policy would not be immediate but would rather be delayed, we excluded two data points (2008–2009) from the analysis and treated 2010 as the initial observation time after policy intervention. The basic model included terms to estimate the baseline level for each variable (intercept), baseline trend (slope), change in the level immediately after the 2007 Artificial Reproduction Act, and changes in trends after the act was implemented.[15–19] We controlled for autocorrelations, because the data used in this study is repeatedly measured monthly over time.[20] To identify the most parsimonious models, we used backward elimination and excluded non-significant terms (P>0.05). Our analysis observed the gaps between the real value and projected value 5 years following the policy implementation. To express the results as a single metric, we also calculated absolute and relative changes (with 95% confidence intervals, CI) [21] 5 years post-policy compared to projected rates. The absolute change is the absolute difference between the regression and the projected regression line in the fifth years following the policy; the relative change is the percentage of the previous difference relative to the projected regression line in the fifth years following the policy. All analyses were carried out with SAS software, Version 9.4 (SAS Institute, Cary, NC).

## Results

We estimated that the rate of unmarried people aged over 15 rose slightly from 34.32% in 1998 to 34.53% in 2007, and to 34.46% in 2017, and the annual growth rate was 0.05%. (Table 1). The average age at time of marriage for females increased from 26.9in 1998 to 29.2 in 2007, and to 31.7 in 2017, with an annual growth rate of 1.11%. The average age of mothers at time of first childbirth increased from 26.4 in 1998 to 28.5 in 2007, and to 30.03 in 2017. The national birth rate declined from 1.23% in 1998 to 0.89% in 2007, and to 0.83% in 2017, with an annual growth rate of -1.39%.

**Table 1:** 1998–2017 trend of basic population data regarding assisted reproductive technology use in Taiwan.

Year	Rate of unmarried people aged over 15	Average female married age	Average mother’s age at time of first childbirth	National birth rate
1998	34.32%	26.90	26.40	1.23%
1999	34.25%	27.00	26.70	1.29%
2000	34.09%	27.00	26.70	1.38%
2001	33.95%	27.40	26.70	1.15%
2002	33.85%	27.90	26.90	1.10%
2003	33.94%	28.40	27.20	1.01%
2004	34.15%	28.00	27.40	0.96%
2005	34.32%	28.50	27.70	0.91%
2006	34.39%	29.00	28.10	0.90%
2007	34.53%	29.20	28.50	0.89%
2008	34.50%	29.50	28.90	0.85%
2009	34.79%	30.30	29.30	0.83%
2010	34.91%	30.50	29.60	0.72%
2011	34.77%	30.60	29.90	0.85%
2012	34.88%	30.84	30.10	1.01%
2013	34.74%	31.03	30.40	0.83%
2014	34.67%	31.25	30.51	0.90%
2015	34.64%	31.39	30.58	0.91%
2016	34.52%	31.50	30.74	0.88%
2017	34.46%	31.70	30.03	0.83%
Linear autoregression model	Y = 0.3424+(0.000168*time)	Y = 26.4556+(0.28*time)	Y = 26.2656+(0.2112*time)	Y = 0.008389+(-0.000109*time)
p-value	0.3626	<0.0001	<0.0001	0.0398
1998–2017 Annual growth rate (%)	0.05%	0.01	0.01	-1.39%

Note: Rate of unmarried people aged over 15: number of unmarried people aged over 15 divided by national population aged over 15 (not including divorced and widowed people); National birth rate: number of births divided by national population aged over 18 in the same year; Annual growth rates were calculated using an autoregressive model.

### Trends in use of assisted reproductive technology

The number of ART treatment cycles is a common metric representing the trend in the use of ART. [4] It more than doubled between 1998 and 2016 (increased from 7,146 in 1998 to 7,941 in 2007, and to 34,486 in 2016), with an annual growth rate of 36.07% (see Table 2).

**Table 2:** 1998–2016 trend of outcomes regarding assisted reproductive technology use in Taiwan.

	Number of ART treatment cycles	Number of births by ART	National number of births	Rate of births by ART
Time	Real value	Real value	Real value	Real value
1998	7,146	2,317	268,881	0.86%
1999	6,966	2,271	284,073	0.80%
2000	7,038	2,358	307,200	0.77%
2001	6,458	2,381	257,866	0.92%
2002	6,622	2,465	246,758	1.00%
2003	5,831	2,270	227,447	1.00%
2004	6,792	2,598	217,685	1.19%
2005	7,346	2,839	206,465	1.38%
2006	7,281	2,793	205,720	1.36%
2007	7,941	2,936	203,711	1.44%
2008	8,354	3,093	196,486	1.57%
2009	9,266	3,464	192,133	1.80%
2010	11,513	4,117	166,473	2.47%
2011	14,645	5,486	198,348	2.77%
2012	16,041	5,825	234,599	2.48%
2013	17,393	5,988	194,939	3.07%
2014	22,684	6,857	211,399	3.24%
2015	29,720	8,254	213,093	3.87%
2016	34,486	8,988	207,600	4.33%
Linear autoregression model	Y = 2432+(1495*time)	Y = 1249+(367.7421*time)	Y = 266085+(-4087*time)	Y = 0.002471+(0.001904*time)
p-value	0.0024	0.0004	0.0337	<0.0001
1998–2016 Annual growth rate (%)	36.07%	0.22	-0.01	41.23%

Note: ART: Assisted Reproductive Technology; Rate of births by ART: number of births by ART divided by national number of births in the same year; Annual growth rates were calculated using an autoregressive model.

In 1998, the number of births by ART was 2,317, which accounted for 0.86% of the national total number of births (268,881) in the same year, and there were 2,936 births by ART in 2007, accounting for 1.44% of national births. The number of births by ART rose substantially to 8,988 in 2016, accounting for 4.33% of the national total number of births (207,600) in the same year, with a 41.23% annual growth rate of the birth rate by ART.

### Impacts of the 2007 Artificial Reproduction Act on use of ART

**Fig 1:**
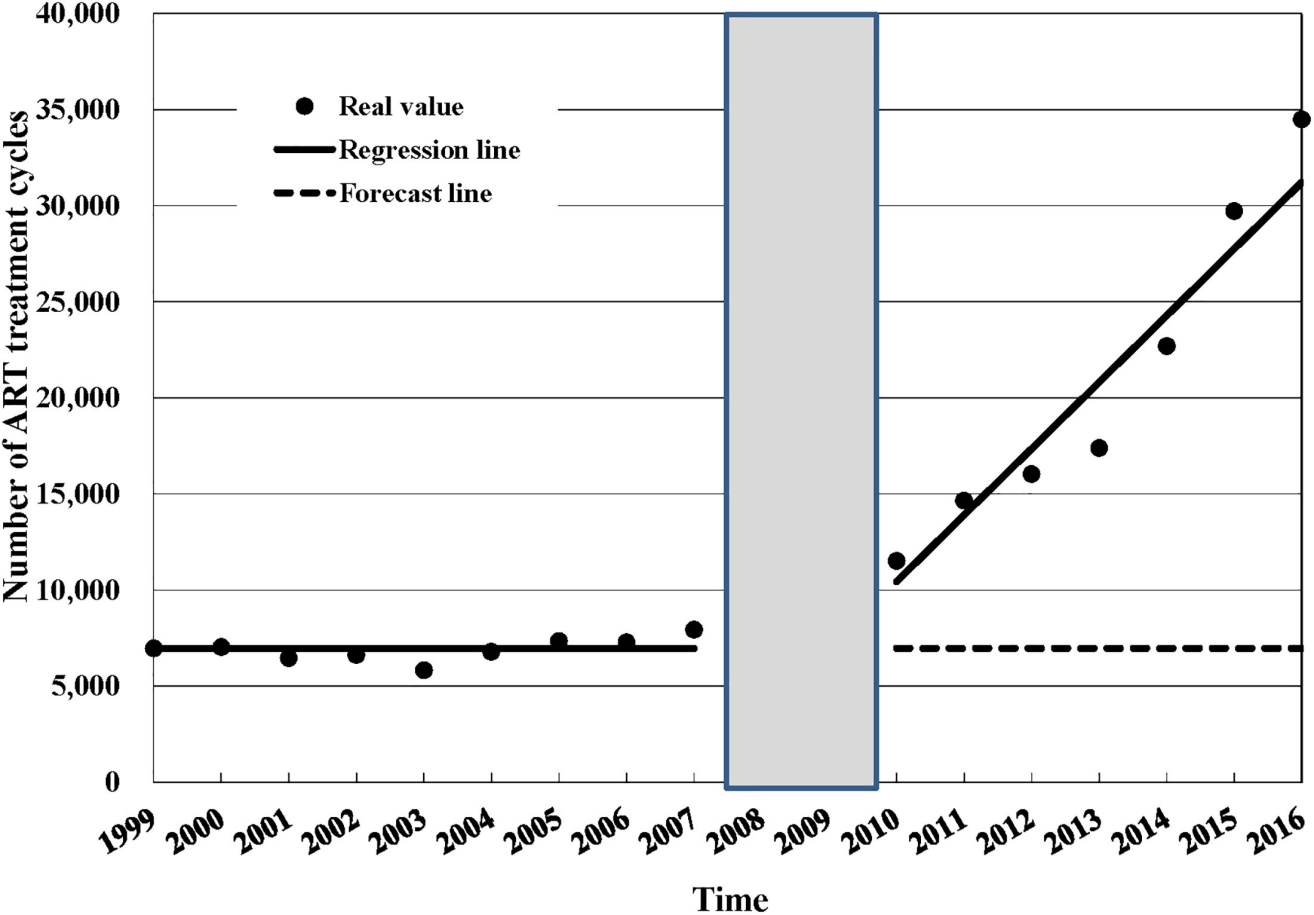
Annual number of ART treatment cycles, 1999–2016. ART = assisted reproductive technology; Since the effect of the policy would not be immediate but would rather be delayed, we excluded two data points (2008–2009) from the analysis and treated 2010 as the initial observation time after policy intervention.

**Fig 2:**
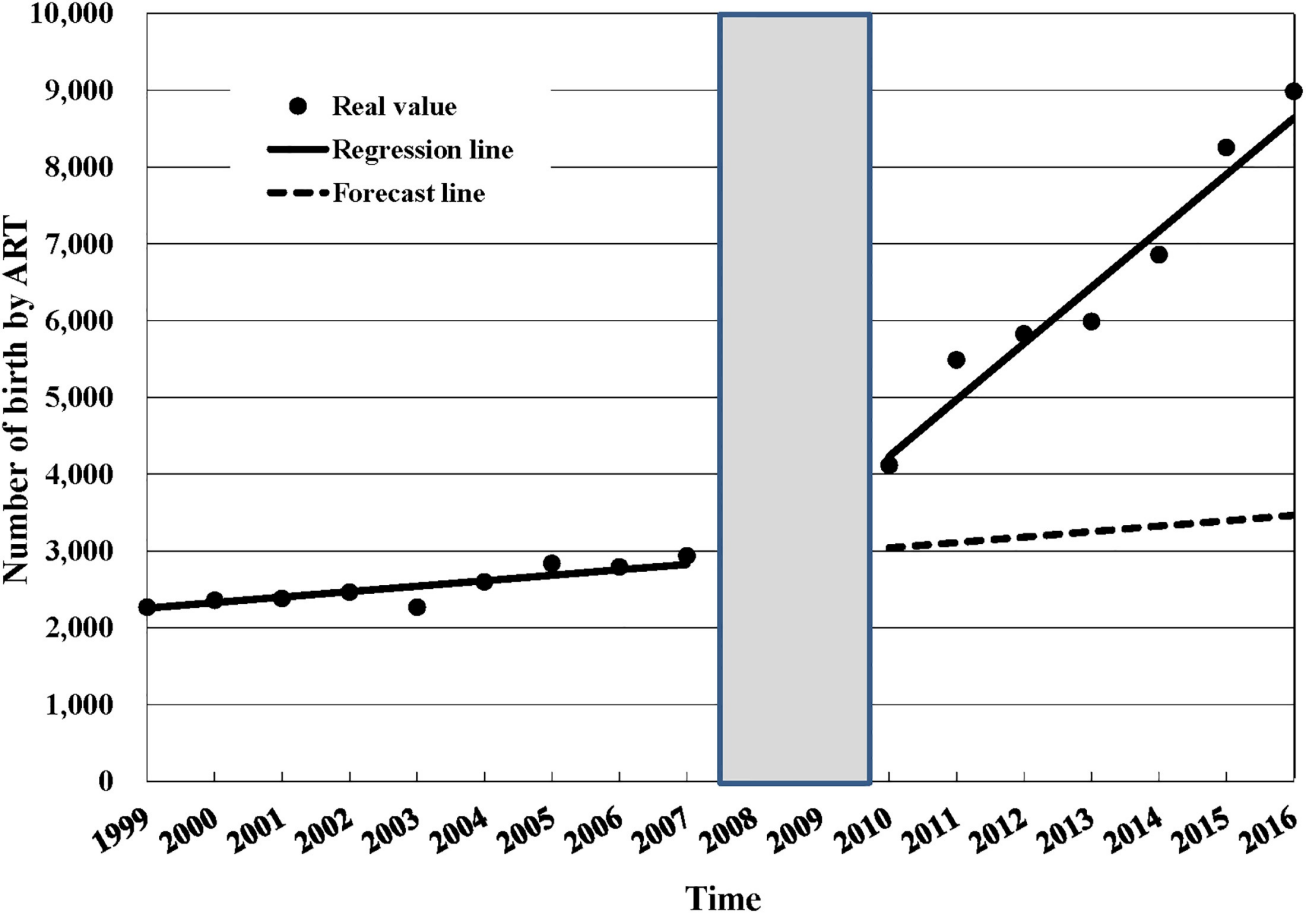
Annual number of births by ART, 1999–2016. ART = assisted reproductive technology; Since the effect of the policy would not be immediate but would rather be delayed, we excluded two data points (2008–2009) from the analysis and treated 2010 as the initial observation time after policy intervention.

**Fig 3:**
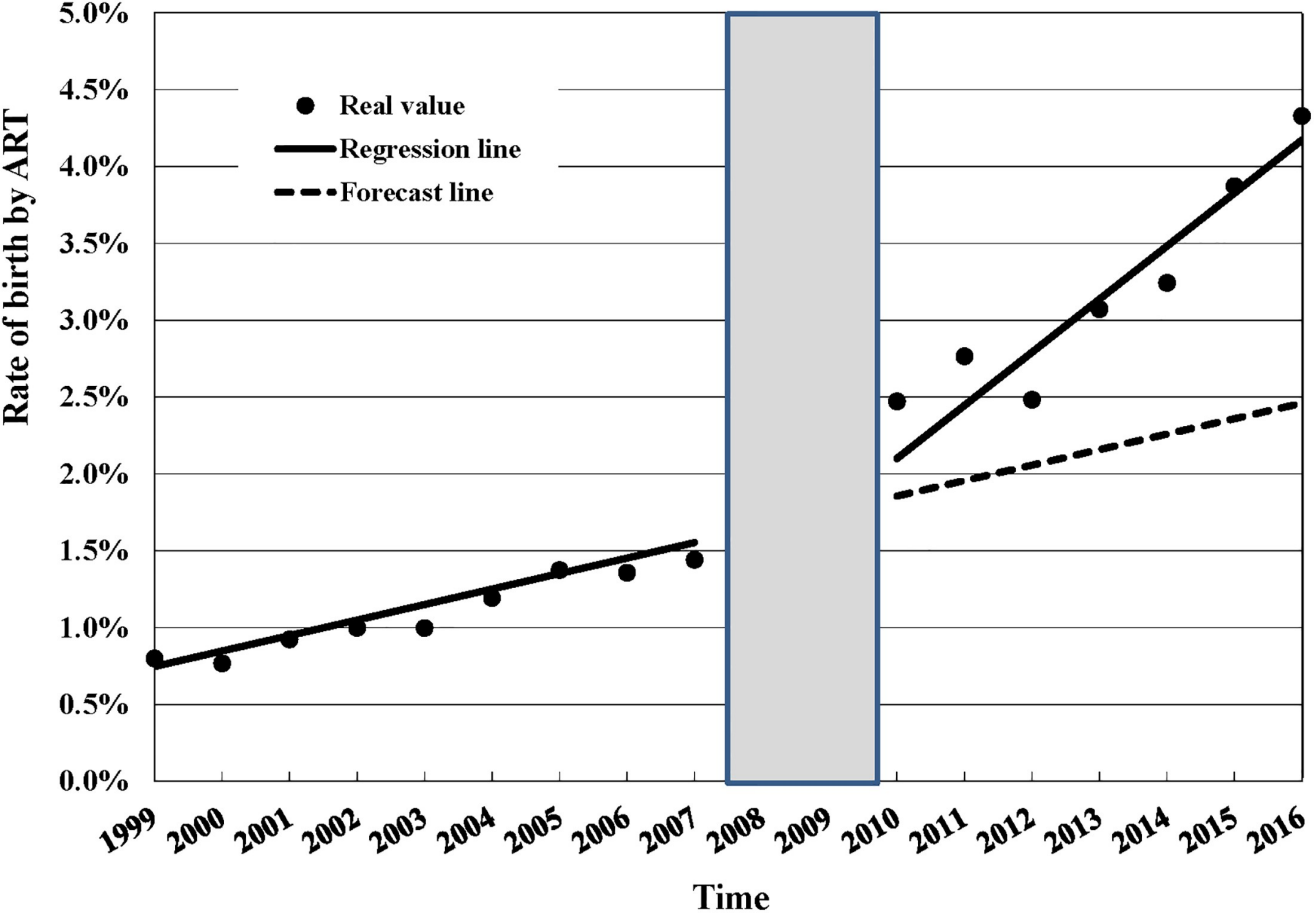
Annual birth rate by ART, 1999–2016. ART = assisted reproductive technology; Rate of births by ART (%) = number of births by artificial reproduction / total number of births; Since the effect of the policy would not be immediate but would rather be delayed, we excluded two data points (2008–2009) from the analysis and treated 2010 as the initial observation time after policy intervention.

Table 3 shows the estimated changes in the rate of ART use and rate of births by ART following the 2007 Artificial Reproduction Act. Three years after the act was implemented, there was a relative increase of 157.8% (95%CI: 127.72%, 187.88%) in the number of ART treatment cycles and a relative increase of 78.51% (95%CI: 46.13%, 110.90%) in the number of births by ART the fifth year following the implementation of the ARA in 2007. The rate of births by ART also increased 35.67% (18.87%, 52.47%) after the 2007 ARA was passed. Figs 1, 2 and 3 show annual number of ART treatment cycles, annual number of births by ART and annual birth rate by ART over time respectively.

**Table 3:** Estimated changes in rate of ART use and rate of births by ART following 2007 Artificial Reproduction Act based on an interrupted time series design and segmented regression models.

	Intercept	Baseline trend	Level change	Trend change	Absolute change (5 year later)	Relative change (5 year later)
Number of ART treatment cycles	6,967	NS	NS	3463 (3238, 3688)	10,737.96 (9,823.70, 11,652.22)	157.80% (127.72%, 187.88%)
Number of births by ART	2,115	71.01 (43.87, 98.15)	532.37 (258.35, 806.39)	662.27 (600.24, 724.31)	2,527.02 (1,915.16, 3,138.88)	78.51% (46.13%, 110.90%)
Rate of births by ART (%)	0.0055	0.0010 (0.0007, 0.0013)	NS	0.0024 (0.0016, 0.0033)	0.0073 (0.0049, 0.0098)	35.67% (18.87%, 52.47%)

Note: ART: Assisted Reproductive Technology; Rate of births by ART: number of births by ART divided by national number of births in the same year; NS: non-significant; 95%CI = estimate +/- (1.96*se); All terms p<0.05 retained in models.

## Discussion

This study examined the past trend in use of ART in Taiwan using a time series analysis with an autoregressive model. This study also used rigorous quasi-experimental study methods to assess the impact of the 2007 Artificial Reproduction Act on use of ART and birth rates by ART. The findings can be used to inform policy makers on strategies and policy development to address low birth rates and infertility in Taiwan. Our study exemplifies an approach to evaluating the impacts of policy changes such as the 2007 Artificial Reproduction Act.

Our study showed that female marriage age and mothers’ age at the time of first childbirth have increased over time in Taiwan and that the national birth rate declined over the same period, dropping to 0.83% in 2017. These trends are similar to those recorded by the Organisation for Economic Co-operation and Development (OECD) countries. The average total fertility rate was merely 1.7% in most OECD countries in 2013, and there were more than 22 countries with an average birth age of more than 30 years old. [22]

We found that use of ART and rate of births by ART increased substantially after the 2007 Artificial Reproduction Act. Compared with the rules governing the use of ART in other countries, Taiwan did not implement specific age restrictions or restrictions on the number of ART treatment cycles, minimizing access barriers and increasing the chances of receiving ART in Taiwan. However, in contrast to some countries such as the United Kingdom [23] that allow all cohabitating partners with or without a marital relationship to receive ART, only legal husbands and wives are allowed to receive ART in Taiwan. Open discussion is needed about the rights of ART for cohabiting partners with or without a marital relationship and single women or men in Taiwan. Furthermore, surrogate motherhood is illegal in Taiwan, similar to countries including Germany [24, 25], France [26, 27], Japan [28] and Singapore [29]. In contrast, surrogate motherhood is legal for non-commercial purposes in some countries including the United Kingdom [23, 30, 31], Australia [32–36], Canada [37, 38] and the Netherlands [39], and it is even allowed for commercial purposes in many Asian countries, including South Korea [28] and India [40, 41]. The appropriateness of the surrogate motherhood policy in Taiwan is also an important issue to be considered.

Following the implementation of the Artificial Reproduction Act in 2007, another “artificial reproduction allowance policy” was implemented in Taiwan in 2015. Public funding from the health and welfare tax on tobacco is used to support access to artificial reproduction for lower income couples with infertility problems. [42] This allowance policy was designed for 3 stages (taking 3 years). In the first year of policy implementation, only low income or low- and middle-income households (determined by average monthly income per person as well as assets such as property and real estate, adjusted for regional differences) were beneficiaries, and the highest financial allowance was around US$3,000. After that, those earning less than 70% and 130% of the average family income per household could apply for the allowance in the second and the third years, respectively. In terms of the targeted people eligible for financial support, studies have shown that government allowances can effectively narrow the economic gap between supply and demand [43], and that economic barriers comprise a powerful factor in acceptance of ART treatment. [44, 45] Providing financial support to only low-income people may not completely improve the fertility rate. It would be important to assess government spending on ART and review ART subsidy policies.

There are some differences in restrictions on artificial reproduction allowance policies between Taiwan and many other countries. Some other countries have specific eligibility requirements for financial support for artificial reproduction [46], such as age (for example, ≦43 years old in France [47]), gender (only female), years after marriage (for example, 2 years after marriage in France [47]), waiting period (for example, 2–3 years in Switzerland [48]), number of treatment cycles, and specific institutions [49]. In comparison, Taiwan has allowed broad access without implementing these restrictions used in other countries; thus, there should be higher motivation to use ART in Taiwan.

The government should not rely solely on ART to increase national fertility. Other policies should be implemented at the same time. The most common approach is to provide economic incentives, including parental benefits and tax reductions, which exclude economic barriers related to fertility. [50] Social support is another incentive to enhance fertility. [51] In the modern popular double-income family models, adequate child care and the ability to stay in the workforce are major issues. Government and employers should consider policies and adequate support for parents, for example, nursery availability, childcare policies, adequate and/or flexible working hours, and systems for transitioning back to work after maternity leave.

Even though ART can improve the chances of pregnancy, allow infertile couples to realize their desire to have children, and improve the possible low fertility problem caused by late marriage or late pregnancy, there are still many shortcomings in the use of ART. In addition to increasing the proportion of multiple births [52, 53], ART is associated with "ovarian hyperstimulation syndrome (OHSS)" [54, 55], patients may have following symptoms: reduced urination, shortness of breath, abdominal pain, rapid weight gain, and blood clots. [55] In addition, when extracting eggs, it may also cause bleeding and infection. Some people are allergic to ovulation drugs and lutein, causing mild skin irritation.[56, 57] Further, ART may be associated with psychological symptoms [58–60], especially after the failure of ART [61]. In addition to the enormous medical expenses, couples who have infertility problems face psychological trauma that could lead to mental illness such as depression. The above-mentioned consequences of ART may require further research and consideration in policy planning.

There are some limitations to this study. First, this study examined the recent trends in the use of ART in Taiwan. We did not analyze the longitudinal trends in light of other possible factors influencing the use of ART, such as technological advances, changes in social values, and changes in physicians’ and patients’ perspectives of ART. These should be examined in future studies. Second, this study did not examine the use of various types of ART for specific types of infertility due to a lack of detailed clinical information. Third, we used a specific age cut-off for some variables based on the available data, for example, the rates associated with unmarried people aged over 15. Finally, the predicted values of ART use were estimated using an economic simulation method (time series design with ARIMA models) and were based on the recent trends; these estimates might not represent real values.

## Conclusion

In Taiwan, the unmarried ratio, the average female age at marriage, and the age at time of first childbirth have gradually increased, and the national birth rate has declined rapidly in the past 19 years. In view of the long-term trend, the problem of future population imbalance will become increasingly serious. On the other hand, this study found that the use of ART has gradually increased, especially after the implementation of the 2007 ARA. ART use can be considered one of the solutions for Taiwan’s late marriage and minority fertility issues.

## Abbreviations

ARA: Artificial Reproduction Act
ARIMA: autoregressive integrated moving average
ART: Assisted Reproduction Technology
NS: non-significant
OECD: co-operation and Development
